# World Trade Center Disaster and Asthma Type: Thomas et al. Respond

**DOI:** 10.1289/ehp.12384R

**Published:** 2009-02

**Authors:** Pauline A. Thomas, Robert Brackbill, Lisa Thalji, Sharon Campolucci, Laura DiGrande, Lorna Thorpe, Steven D. Stellman, Kelly Henning

**Affiliations:** New Jersey Medical School–UMDNJ, Newark, New Jersey, E-mail: Thomasp1@umdnj.edu; Agency for Toxic Substances and Disease Registry, U.S. Department Health and Human Services, Atlanta, Georgia; RTI International, Chicago, Illinois; New York City Department of Health and Mental Hygiene, New York, New York; Bloomberg Foundation, New York, New York

We appreciate the question raised by Ziem regarding the type of asthma reported in children exposed to the World Trade Center (WTC) disaster of 11 September 2001 (9/11) (Thomas et al. 2008). In our study we found an increase in age-specific asthma prevalence among children < 12 years of age, and a new diagnosis of asthma was strongly correlated with a report of exposure to the dust and debris cloud that occurred as buildings collapsed on 9/11. Among children < 5 years of age, we observed an increased prevalence in asthma diagnosis even among those not exposed to the dust cloud.

We cannot confirm or disprove Ziem’s very reasonable suggestion that the asthma seen here was irritant rather than allergen induced. The data on asthma diagnoses were reported by parents or other guardians of the children, using a very simple standardized question. We asked whether a medical provider had ever said the child had asthma, and if yes, whether this occurred before or after 9/11. We did not collect information on severity, treatment, or duration, and did not review medical records. A follow-up survey of the children is under way and includes questions to characterize the asthma illness, but those data are not yet available.

In small children, in addition to airborne irritants and atopy, respiratory viruses may also play a role in initiation or exacerbation of asthma (Schwarze and Gelfand 2000). It is difficult or impossible in a general pediatric setting to differentiate the type of asthma in children, and many pediatricians use the term “reactive airway disease” in children with recurrent wheezing whether or not there appears to be an allergic component.

The airborne contaminants immediately following the 9/11 attacks included a highly alkaline mixture of gypsum, concrete, and synthetic vitreous fibers, further contaminated by metals, organochlorine compounds such as polychlorinated biphenyls and dioxins, and polycylic aromatic hydrocarbons. Later on, settled dust in indoor environments, including residences where many children lived, was found to consist of both fine, coarse, and “supercoarse” (> 10 μm in diameter) particulate matter (Lioy et al. 2006); thus, there was a clear potential for exposure to biologic or allergenic substances. Also, as the cleanup for homes, as well as public areas, was prolonged and difficult, molds could have occurred in some environments and contributed to allergic reactions.

Based on earlier studies, Landrigan et al. (2004) noted that “high alkalinity of WTC dust produced bronchial hyperreactivity, persistent cough, and increased risk of asthma.” Increased asthma was subsequently reported in evaluations of 68,444 adults enrolled in the WTC Health Registry (Farfel et al. 2008). Wheeler et al. (2007) found a dose response with increased exposure to the 16-acre “pile” of debris associated with the buildings’ collapse and burning. In an overview of health effects in other adults, Farfel et al. (2008) found an association of asthma with exposure to the initial dust cloud generated by the collapse of the twin towers.

We agree with Ziem that, in both children and adults, the exposures observed are more likely related to particulates and other irritants. Further work that includes more detailed histories accompanied by pulmonary function testing is needed to better characterize the pulmonary illness in these individuals.
